# Supplementary material for: Impact of intraoperative transesophageal echocardiogram on changes in surgical management among patients undergoing cardiovascular surgery in Thailand
Source: PLoS One. 2026 Jan 20;21(1):e0341156. doi: 10.1371/journal.pone.0341156 (PMC12818624; doi:10.1371/journal.pone.0341156)
Supplement: S1 Table — (PDF) [file pone.0341156.s001.pdf]

S1 Table. Waiting time intervals between TTE alone and TTE combined with TEE groups by surgical management change status due to intraoperative TEE.

Group	Change in management due to intraoperative TEE	Preoperative Echocardiogram	n	From TTE to the operative date Median (IQR)	p-value	From the last imaging to the operative date Median (IQR)	p-value
Overall	Yes	TTE+TEE	15	216 (84, 299)	0.022*	171 (35, 223)	0.171
		TTE alone	51	58 (12, 170)		58 (12, 170)	
	No	TTE+TEE	78	194 (91, 299)	<0.001*	152.5 (66, 234)	<0.001*
		TTE alone	480	76 (11, 188)		76 (11, 188)	
Elective	Yes	TTE+TEE	14	209 (84, 280)	0.058	132 (35, 218)	0.372
		TTE alone	49	65 (12, 170)		65 (12, 170)	
	No	TTE+TEE	78	194 (91, 299)	<0.001*	152.5 (66, 234)	0.005*
		TTE alone	426	98 (24, 201)		98 (24, 201)	

* p-value<0.05
