# Supplementary material for: Impact of intraoperative transesophageal echocardiogram on changes in surgical management among patients undergoing cardiovascular surgery in Thailand
Source: PLoS One. 2026 Jan 20;21(1):e0341156. doi: 10.1371/journal.pone.0341156 (PMC12818624; doi:10.1371/journal.pone.0341156)
Supplement: S2 Table — (PDF) [file pone.0341156.s002.pdf]

S2 Table. Waiting time intervals between surgical management change statuses due to intraoperative TEE, stratified by preoperative echocardiography subgroups

Preoperative Echocardiogram	Change in management due to intraoperative TEE	n	From TTE to the operative date Median (IQR)	p-value	From the last imaging to the operative date Median (IQR)	p-value
all	Yes	66	88.5 (15, 216)	0.853	75 (13, 202)	0.7223
	No	558	95.5 (14, 211)		94 (14, 198)	
TTE alone	Yes	51	58 (12, 170)	0.251	58 (12, 170)	0.2513
	No	480	76 (11, 188)		76 (11, 188)	
TTE+TEE	Yes	15	216 (84, 299)	0.622	171 (35, 223)	0.4560
	No	78	194 (91, 299)		152.5 (66, 234)	
