# Supplementary material for: Impact of intraoperative transesophageal echocardiogram on changes in surgical management among patients undergoing cardiovascular surgery in Thailand
Source: PLoS One. 2026 Jan 20;21(1):e0341156. doi: 10.1371/journal.pone.0341156 (PMC12818624; doi:10.1371/journal.pone.0341156)
Supplement: S3 Table — (PDF) [file pone.0341156.s003.pdf]

S3 Table. Sensitivity analyses for changes in surgical management due to any causes: Crude and adjusted RR

Variables	Yes n = 166 (26.60%)	No n = 458 (73.40%)	Crude RR (95%CI)	p-value	Adjusted RR (95%CI)	p-value
Preoperative TEE						
Yes (TTE + TEE)	130 (24.48)	401 (75.52)	1.58 (1.18, 2.13)	0.002*	1.27 (0.94, 1.72)	0.116
No (TTE alone)	36 (38.71)	57 (61.29)	1		1	
Sex						
Male	96 (26.89)	261 (73.11)	1.03 (0.79, 1.34)	0.851	1.08 (0.84, 1.39)	0.543
Female	70 (26.22)	197 (73.78)	1		1	
Age						
Less than 60 years	67 (32.21)	141 (67.79)	1	0.023*	1	0.812
At least 60 years	99 (23.80)	317 (76.20)	0.74 (0.57, 0.96)		0.97 (0.75, 1.26)	
Status						
Elective	150 (26.46)	417 (73.54)	1	0.791	1	0.186
Urgency/Emergency	16 (28.07)	41 (71.93)	1.06 (0.68, 1.64)		0.73 (0.46, 1.16)	
Type of Surgery						
(1) CABG	8 (4.94)	154 (95.06)	1	-	1	-
(2) Valve surgery	76 (32.62)	157 (67.38)	6.61 (3.28, 13.31)	<0.001*	6.04 (2.96, 12.34)	<0.001*
(3) Thoracic-aortic surgery	19 (42.22)	26 (57.78)	8.55 (4.01, 18.23)	<0.001*	9.75 (4.47, 21.29)	<0.001*
(1) + (2)	23 (26.44)	64 (73.56)	5.35 (2.50, 11.46)	<0.001*	5.37 (2.51, 11.48)	<0.001*
(1) + (3)	4 (57.14)	3 (42.86)	11.57 (4.56, 29.38)	<0.001*	11.07 (4.32, 28.39)	<0.001*
(2) + (3)	19 (57.58)	14 (42.42)	11.66 (5.58, 24.35)	<0.001*	11.56 (5.49, 24.30)	<0.001*
(1) + (2) + (3)	4 (44.44)	5 (55.56)	9.00 (3.33, 24.34)	<0.001*	8.87 (3.28, 24.01)	<0.001*
ASD/VSD	2 (13.33)	13 (86.67)	2.70 (0.63, 11.58)	0.181	2.32 (0.52, 10.32)	0.271
Cardiac mass removal	3 (33.33)	6 (66.67)	6.75 (2.15, 21.20)	0.001*	7.31 (2.33, 22.91)	0.001*
Other	8 (33.33)	16 (66.67)	6.75 (2.80, 16.29)	<0.001*	6.36 (2.62, 15.51)	<0.001*
Location of TTE						
In house	140 (27.45)	370 (72.55)	1	0.321	1	0.783
Outside	26 (22.81)	88 (77.19)	0.83 (0.58, 1.20)		0.95 (0.68, 1.34)	

* p-value < 0.05
