# Supplementary material for: Impact of intraoperative transesophageal echocardiogram on changes in surgical management among patients undergoing cardiovascular surgery in Thailand
Source: PLoS One. 2026 Jan 20;21(1):e0341156. doi: 10.1371/journal.pone.0341156 (PMC12818624; doi:10.1371/journal.pone.0341156)
Supplement: S4 Table — (PDF) [file pone.0341156.s004.pdf]

S4 Table. Waiting time intervals between TTE alone and TTE combined with TEE groups by surgical management changes due to any causes.

Group	Change in management due to any causes	Preoperative Echocardiogram	n	From TTE to the operative date Median (IQR)	p-value	From the last imaging to the operative date Median (IQR)	p-value
Overall	Yes	TTE+TEE	36	217 (88.5, 299)	<0.001*	186.5 (54.5, 236.5)	0.003*
		TTE alone	129	49.5 (6, 177)		49.5 (6, 177)	
	No	TTE+TEE	57	189 (91, 286)	<0.001*	150 (66, 206)	0.004*
		TTE alone	401	81 (13, 188)		81 (13, 188)	
Elective	Yes	TTE+TEE	35	216 (84, 299)	0.002*	171 (47, 234)	0.046*
		TTE alone	115	76 (21, 198)		76 (21, 198)	
	No	TTE+TEE	57	189 (91, 286)	<0.001*	150 (66, 206)	0.036*
		TTE alone	360	98.5 (27, 199.5)		98.5 (27, 199.5)	

* p-value<0.05
