# Supplementary material for: Impact of intraoperative transesophageal echocardiogram on changes in surgical management among patients undergoing cardiovascular surgery in Thailand
Source: PLoS One. 2026 Jan 20;21(1):e0341156. doi: 10.1371/journal.pone.0341156 (PMC12818624; doi:10.1371/journal.pone.0341156)
Supplement: S5 Table — (PDF) [file pone.0341156.s005.pdf]

S5 Table. Waiting time intervals between surgical management change statuses due to any cause, stratified by preoperative echocardiography subgroups

Preoperative Echocardiogram	Change in management due to any causes	n	From TTE to the operative date Median (IQR)	p-value	From the last imaging to the operative date Median (IQR)	p-value
all	Yes	166	92 (11, 223)	0.739	83.5 (11, 212)	0.968
	No	458	96.5 (16, 202)		94.5 (16, 190)	
TTE alone	Yes	130	49.5 (6, 177)	0.971	49.5 (6, 177)	0.971
	No	401	81 (13, 188)		81 (13, 188)	
TTE+TEE	Yes	36	217 (88.5, 299)	0.950	186.5 (54.5, 236.5)	0.770
	No	57	189 (91, 286)		150 (66, 206)	
