# Supplementary material for: Impact of intraoperative transesophageal echocardiogram on changes in surgical management among patients undergoing cardiovascular surgery in Thailand
Source: PLoS One. 2026 Jan 20;21(1):e0341156. doi: 10.1371/journal.pone.0341156 (PMC12818624; doi:10.1371/journal.pone.0341156)
Supplement: Attachment — Submitted filename: Response to Reviewers.docx [file pone.0341156.s006.docx]

Dear Wei Wu,

I hope this message finds you well. We are pleased to submit the revised version of our manuscript entitled “Impact of Perioperative Transesophageal Echocardiogram and Preoperative Echocardiography Waiting Time on Surgical Management in Thailand” (Manuscript Number: **PONE-D-25-35063**), following the helpful and insightful comments provided by you and the reviewers. We would like to express our sincere gratitude for the constructive feedback, which has significantly improved the quality of our work.

After thorough discussion with our team, we have categorized the comments into items, as outlined below.

**Editor’s comments:**

1. Although the manuscript suggests that intraoperative TEE was performed in all included cases, this point is not consistently or clearly stated. Given that the primary outcome relates directly to TEE’s influence on surgical planning, it is essential to clearly document the TEE rate and whether it was applied uniformly to all patients. In addition, the manuscript would benefit from more clarity on patient selection, flow, and subgroup structure. For example, it remains somewhat confusing how the subgroups (e.g., TTE-only, TEE-only, or both) were formed, and whether TEE indications were predefined or at the discretion of the care team.

**Response:** We have revised the manuscript to state explicitly that intraoperative TEE was performed on a cohort representing 74.29% of all cardiac surgeries during the study period; this is now clarified in the first paragraph of the Results section and in the inclusion/exclusion criteria. We have also refined the Methods section under "Study design and subjects" to define the indications for intraoperative TEE, noting that the decision was based on a joint agreement between the attending cardiac anesthesiologist and surgeon.

To address the confusion regarding patient groups, we have clarified that the "TTE-only" and "TTE+TEE" designations refer to *preoperative* imaging assessments and were analyzed as factors of interest, not as prospectively formed study groups. The revised Figure 1 (Study Flow Diagram) now more clearly illustrates patient selection and flow. Finally, we have standardized the terminology to "intraoperative TEE" throughout the manuscript for consistency.

**2.** The lack of postoperative outcome analysis limits the ability to fully assess the clinical impact of TEE-guided surgical plan changes. While this may have been outside the original scope, the inclusion of even limited outcome data (e.g., 30-day mortality, complication rates, or length of stay) would substantially strengthen the conclusions and may be feasible from prospective dataset.

**Response:** We appreciate and agree completely with this valuable suggestion. We have now incorporated a comprehensive analysis of postoperative outcomes through September 2025. This new dataset includes the duration of mechanical ventilation, length of ICU and hospital stays, 30-day mortality, and the incidence of major complications (e.g., atrial fibrillation, reoperation, stroke, acute kidney injury). The Results and Discussion sections have been substantially revised to integrate these findings, providing a more robust assessment of the clinical implications of TEE-guided management changes.

**Reviewer’s comments**

1. Is the manuscript technically sound, and do the data support the conclusions?

Reviewer #1: Partly

**Response:** With the addition of the comprehensive postoperative outcomes analysis and the clarifications made in response to all editor and reviewer comments, we believe the manuscript is now technically sound and that the conclusions are more robustly supported by the data.

2. Has the statistical analysis been performed appropriately and rigorously?

Reviewer #1: No

**Response:** We have re-examined our methodology and confirm that standard, appropriate statistical methods were applied, consistent with the study's prospective observational design and data characteristics, as detailed in the Statistical Analysis section. Based on our best knowledge, we did it appropriately but would be grateful for any specific suggestions for further improvement.

3. Have the authors made all data underlying the findings in their manuscript fully available?

Reviewer #1: Yes

4. Is the manuscript presented in an intelligible fashion and written in standard English?
Reviewer #1: Yes

5. Review Comments to the Author

**Reviewer #1:** # Summary
1. These investigators conducted a prospective observational study to investigate the impact of intraoperative TEE on surgical planning (primary outcome) and wait times (secondary outcome), among 624 cardiac surgical patients undergoing surgery the calendar year of 2023. They found intraoperative TEE altered the surgical plan in 10% of cases, but did not study clinical outcomes.

\# Overall Assessment
1. This is important work on the impact of TEE in a resource-limited situation. However, the absence of investigating TEE's impact on postsurgical outcomes limits the impact and makes this study largely descriptive.
**Response:** As detailed in our response to the editor (Comment #2), we have now conducted and integrated a full analysis of postoperative clinical outcomes. This addition moves the study beyond a descriptive focus and provides crucial data on the clinical impact of TEE-guided decisions, significantly enhancing the manuscript's contribution.

\# Major Issues / Questions
1. what was the overall rate of intraoperative TEE? I see the rate of preoperative TEE was only 15% but I don't see intraoperative TEE reported. If intraoperative TEE was performed in only 15% of cases, there is a major selection bias and residual confounding is likely present. At a minimum matching would be necessary and could consider 1:2 or even 1:3 matching if TEE rate is truly only 15%.

**Response:** We apologize for the lack of clarity. The 15% rate referred to preoperative TEE. We have now clarified in the Methods and Results sections, as well as in the revised Figure 1, that intraoperative TEE was performed in 624 patients, constituting 74.29% of all cardiac surgeries during the study period. This high rate of utilization strengthens the validity of our findings regarding its impact.

2. While a change in surgical plan is important, it would be stronger if there were an associated postoperative outcomes analysis (e.g. lower rates of reoperation, stroke, complications, death, etc. with TEE)?

**Response:** As stated in response to editor’s comments and overall assessment of the reviewer’s comments.

\# Minor Issues
1. The terminology needs to be consistent. Decide on "perioperative" or "intraoperative" TEE and keep it consistent throughout.

**Response:** We thank the reviewer for pointing this out. The manuscript has been revised to use the term "intraoperative TEE" consistently throughout.

2. Consider limiting the analysis to intraoperative TEE (instead of adding TTE and the wait times). It starts to become confusing to the reader to interpret.

**Response:** To streamline the main text, we have moved the detailed results and tables related to preoperative imaging and waiting times into the Supplementary Information. The primary manuscript now focuses more sharply on the impact of intraoperative TEE, while still retaining the waiting time discussion to provide essential context regarding resource limitations in our setting.

6. PLOS authors have the option to publish the peer review history of their article ([what does this mean?](https://track.editorialmanager.com/CL0/https:%2F%2Fjournals.plos.org%2Fplosone%2Fs%2Feditorial-and-peer-review-process%23loc-peer-review-history/1/010f019914458b69-836c72bd-c55c-4bd2-9b0d-0ea6ae228837-000000/hWqyPud4FnCcrlznU1bzejgXoFWVIIwhUWxvjbLiiak=227)). If published, this will include your full peer review and any attached files.

Reviewer #1: No

We believe that these revisions have strengthened the manuscript, and we hope that the revised version meets the expectations of both you and the reviewers. We look forward to your feedback and hope for a favorable consideration of our submission for publication in PLOS ONE.

Thank you again for your time and consideration.

Sincerely,

Naruenart Lomarat
