# Supplementary material for: Impact of intraoperative transesophageal echocardiogram on changes in surgical management among patients undergoing cardiovascular surgery in Thailand
Source: PLoS One. 2026 Jan 20;21(1):e0341156. doi: 10.1371/journal.pone.0341156 (PMC12818624; doi:10.1371/journal.pone.0341156)
Supplement: Attachment — Submitted filename: Response_to_Reviewers_auresp_2.docx [file pone.0341156.s007.docx]

**Dear Wei Wu,**

We are pleased to submit the revised version of our manuscript, “Impact of Intraoperative Transesophageal Echocardiogram on Changes in Surgical Management among Patients Undergoing Cardiovascular Surgery in Thailand” (Manuscript Number: **PONE-D-25-35063R1**). We would like to express our sincere gratitude for the constructive feedback and helpful comments provided, which have significantly improved the quality of our work

**Editor’s comments:**

1, The Discussion suggests that TEE-guided changes may have mitigated higher intrinsic surgical risk and therefore resulted in similar postoperative outcomes between groups. This is a causal inference that is not supported by the study design, because all patients received intraoperative TEE and no control group exists for comparison. Please rephrase sentences to avoid implying that intraoperative TEE reduced postoperative risk.

**Response:** Thank you for raising this important and subtle point, reflecting the meticulous review provided. To address this issue, we have carefully revised the Discussion to adopt a more conservative tone, focusing on associations rather than causal links. We have also explicitly stated the limitations of interpreting these results due to the observational study design and the absence of a control group for comparison, both in the Discussion and the Limitations section.

**Changes in the Manuscript:**

**From:** “TEE-guided interventions likely identified and corrected unanticipated findings in patients with more complex or severe pathology, thereby mitigating higher inherent risks and yielding outcomes similar to those in the less complex “no-change” group.”

**To:** “Intraoperative TEE facilitated the identification of unanticipated findings in patients with complex or severe pathology. While postoperative outcomes were similar between the 'change' and 'no-change' groups, the observational study design and the universal use of intraoperative TEE preclude a direct assessment of whether these modifications prevented adverse outcomes.”

Added to the final paragraph of the Discussion: “Furthermore, the current study was not designed to establish a direct causal link between these modifications and a reduction in postoperative risk, or it may be that such benefits are not fully reflected in short-term postoperative metrics.”

2, Although the TTE+TEE group had significantly longer preoperative waiting times, the manuscript states that “disease severity may evolve during the waiting period.” However, the data do not show that waiting time independently contributed to surgical management changes. Please clarify the interpretation by avoiding language that implies a causal relationship (e.g., “waiting time leads to disease progression”). This is important for preventing overinterpretation.

**Response:** We appreciate this guidance to avoid overinterpretation of the relationship between waiting times and clinical evolution. We have revised this section of the Discussion to ensure our conclusions remain strictly aligned with our data. Specifically, we have removed language implying a causal link between waiting time and disease progression and instead report the findings as a lack of independent association in our multivariable analysis.

**Changes in the Manuscript:**

**From:** “These findings may suggest that disease severity and case complexity were already well-accounted for in surgical scheduling. Nonetheless, disease status may continue to evolve during the preoperative period, particularly in complex cases. This assumption is supported by the findings in S2 Table, which show no significant differences in waiting times between patients with and without management changes, in both the TTE-only and TTE with TEE subgroups. Still, the waiting time is a risk factor for morbidity and mortality outcomes after cardiac surgery.”

**To:** “While disease status may potentially evolve during the preoperative period, particularly in complex cases, this data showed no significant difference in waiting times between patients with and without management changes in either the preoperative TTE alone or preoperative TTE combined with TEE subgroups (S2 Table). These findings suggest that, in this study, waiting time was not independently associated with surgical management changes, though it remains a known risk factor for morbidity and mortality after cardiac surgery [19, 20].”

3, Although improved, several sections still contain grammatical issues (like "preoperative TTEalone group"). Please have a final round of careful English editing is recommended to ensure clarity and professionalism.

**Response:** We sincerely appreciate the Editor’s feedback regarding the manuscript's presentation. To ensure a high standard of clarity and professionalism, the entire manuscript has undergone a final round of meticulous English language editing. We have specifically focused on:

- Terminology Consistency: We have standardized all terms throughout the manuscript. The preoperative groups are now consistently referred to as the "preoperative TTE alone group" and the "preoperative TTE combined with TEE group." The intraoperative assessment is consistently termed "intraoperative TEE."
- Typo error: We have corrected all spacing issues (e.g., "TTEalone") and ensured that mathematical symbols and ranges use the appropriate formatting.
- Grammatical Integrity: The manuscript was reviewed for grammatical accuracy using Gemini (Google) for initial identification of potential errors, followed by extensive manual editing and expert review to ensure technical and clinical precision. While we believe the text is now clear and professionally polished, we remain available for further refinement should any minor stylistic points remain.
- We have conducted a thorough audit of the reference list to ensure accuracy, technical consistency, and full compliance with PLOS ONE guidelines. This process included cross-referencing all citations against retraction databases to confirm that no retracted works are included in the manuscript. Additionally, we carefully evaluated all reviewer-suggested literature, incorporating those publications that provided relevant academic context to enhance our study’s discussion. All references have been verified for correct formatting and are accurately cited within the text.

**Reviewer’s comments**

All comments have been adequately addressed

We believe these revisions have significantly strengthened the manuscript and hope the revised version now meets the expectations of the Editor and the reviewers. We look forward to your decision and thank you once again for your time and thoughtful consideration of our work.

Sincerely,

Naruenart Lomarat
